# Can AI-Based ChatGPT Models Accurately Analyze Hand–Wrist Radiographs? A Comparative Study

**DOI:** 10.3390/diagnostics15121513

**Published:** 2025-06-14

**Authors:** Ahmet Yıldırım, Orhan Cicek, Yavuz Selim Genç

**Affiliations:** 1 Department of Orthodontics, Faculty of Dentistry, Zonguldak Bulent Ecevit University, Zonguldak 67600, Türkiye; orhancicek@beun.edu.tr; 2 Samsun Oral and Dental Health Hospital, Samsun Provincial Health Directorate, Samsun 55060, Türkiye; yavuz_selim_genc@hotmail.com

**Keywords:** large language models, artificial intelligence, deep learning, ChatGPT, bone age, growth stage, convolutional neural network

## Abstract

**Background/Aims:** The aim of this study was to evaluate the effectiveness of large language model (LLM)-based chatbot systems in predicting bone age and identifying growth stages, and to explore their potential as practical, infrastructure-independent alternatives to conventional methods and convolutional neural network (CNN)-based deep learning models. **Methods**: This study evaluated the performance of three ChatGPT-based models (GPT-4o, GPT-o4-mini-high, and GPT-o1-pro) in predicting bone age and growth stage using 90 anonymized hand–wrist radiographs (30 from each growth stage—pre-peak, peak, and post-peak—with equal male and female distribution). Reference standards were ensured by expert orthodontists using Fishman’s Skeletal Maturity Indicators (SMI) system and the Greulich–Pyle Atlas, with each radiograph analyzed by three GPT models using standardized prompts. Model performances were evaluated through statistical analyses assessing agreement and prediction accuracy. **Results**: All models showed significant agreement with the reference values in bone age prediction (*p* < 0.001), with GPT-o1-pro having the highest concordance (Pearson r = 0.546). No statistically significant difference was observed in the mean absolute error (MAE) among the models (*p* > 0.05). The GPT-o4-mini-high model achieved an accuracy rate of 72.2% within a ±2 year deviation range for bone age prediction. The GPT-o1-pro and GPT-o4-mini-high models showed bias in the Bland–Altman analysis of bone age predictions; however, GPT-o1-pro yielded more reliable predictions with narrower limits of agreement. In terms of growth stage classification, the GPT-4o model achieved the highest agreement with the reference values (κ = 0.283, *p* < 0.001). **Conclusions**: This study shows that general-purpose GPT models can support bone age and growth stages prediction, with each model having distinct strengths. While GPT models do not replace clinical examination, their contextual reasoning and ability to perform preliminary assessments without domain-specific training make them promising tools, though further development is needed.

## 1. Introduction

In orthodontics, deep learning-based convolutional neural networks (CNNs) are employed across a wide spectrum of applications, ranging from the prediction of growth and developmental stages to diagnosis and treatment planning. However, due to their requirement for high computational power, technical expertise, and specialized datasets, the use of these CNN-based artificial intelligence systems remains largely confined to professional settings. In contrast, chatbot systems developed on the basis of large language models (LLMs) offer significant potential in this field by enabling much broader and more accessible applications of artificial intelligence, owing to their easy availability via the internet and their capacity to perform a wide variety of tasks [1,2].

LLMs are currently experiencing rapid and transformative advancements. Artificial intelligence-based interactive chatbots such as ChatGPT (Generative Pre-Training Transformer; OpenAI, San Francisco, CA, USA), Copilot (Microsoft Corporation, Redmond, WA, USA), Gemini (Google Ireland Limited, Dublin, Ireland), LLaMA (Meta Platforms, Menlo Park, CA, USA), Claude AI (Anthropic, San Francisco, CA, USA), DeepSeek (DeepSeek-VL, Shanghai, China), and Grok (xAI, San Francisco, CA, USA) have significantly reshaped the domain of language-based artificial intelligence [3]. These LLM-based systems rely on intelligent human–computer interaction and are designed to simulate conversational exchanges with human users via internet-based platforms [4]. The capabilities of LLMs are notably impressive; they are able to generate fluent and coherent text, respond to queries in dialogic settings, perform image-based analyses, facilitate cross-linguistic translation, and carry out a wide range of language-related tasks [5]. Typically, LLMs are deep neural networks trained on massive corpora of textual data sourced from the internet, including encyclopedic content (e.g., Wikipedia), digital books, academic articles, and web pages. Their primary function is to generate contextually appropriate and human-like responses based on user input (such as prompts or instructions), utilizing advanced deep learning algorithms and sophisticated modeling architectures [6,7].

In recent years, deep learning models, particularly CNNs, have led to a groundbreaking transformation in medical image analysis [8]. These technologies have demonstrated remarkable success in tasks such as the automated detection of pathologies and the segmentation of anatomical structures [9,10]. While CNNs have achieved notable progress in visual pattern recognition [9], the advent of LLMs, initially developed for natural language processing, has expanded analytical capabilities through the integration of multimodal data. This integration enables the contextual interpretation of visual information within a linguistic framework. Such advancements signal the onset of a new era in healthcare applications and carry the potential to fundamentally redefine diagnostic methodologies in medical imaging [8].

In the fields of orthodontics and dentofacial orthopedics, the timing of treatment initiation is at least as crucial as the selection of an appropriate treatment protocol. Initiating treatment during the patient’s optimal stage of skeletal maturation is associated with more favorable therapeutic outcomes and a reduced risk of complications. From an orthodontic standpoint, skeletal maturity is considered a continuous developmental process, commonly assessed using hand–wrist radiographs [11]. The stages of skeletal growth and development can be identified through the use of the Fishman method on hand–wrist radiographs [12]. The Greulich–Pyle and Tanner–Whitehouse methods are commonly used for assessing bone age from hand–wrist radiographs. [11]. However, these assessment techniques typically involve the manual comparison of multiple radiographic images, which can be time-consuming and subject to inter-observer variability [13]. Therefore, although various computer-aided automated bone age prediction methods capable of achieving high accuracy have been developed [14,15,16], the setup, training, and use of these systems typically require advanced technical expertise, robust hardware infrastructure, and specialized datasets. Table 1 summarizes the studies in the literature that utilize various machine learning algorithms for automated bone age assessment, along with their reported performance metrics, including mean absolute error (MAE), mean squared error (MSE), and root mean square error (RMS).

For this reason, the practical application of such models remains limited. In contrast, chatbot systems powered by LLMs stand out for their ease of use without requiring technical knowledge and for their accessibility via web-based interfaces. In this regard, AI systems based on LLMs have emerged as a significant alternative in clinical decision support processes, particularly in terms of practicality, speed, and accessibility. However, the reliability and quality of web-based information remain critical concerns [21], and when these systems are used for diagnostic purposes, there must be an awareness of the potential for inaccurate diagnoses. With this awareness in mind, recent studies suggest that chatbots may offer support for alternative differential diagnoses [22]. While LLMs investigated for this purpose have shown considerable success in text-based applications, their potential in image analysis has yet to be fully explored. Given that LLMs are capable of integrating visual information within a linguistic context, it is anticipated that they may enable comprehensive analysis of medical images and demonstrate promising performance in radiographic diagnosis [8].

Currently, deep learning-based systems have demonstrated substantial success in various domains of visual analysis, ranging from the detection of interstitial lung disease patterns in chest radiographs [1] to the segmentation of vascular networks in fundus photography [23]. These systems have also achieved high levels of accuracy in predicting bone age from hand–wrist radiographs. However, such approaches involve labor-intensive and technically demanding processes, including extensive data collection, image preprocessing, model training, and hyperparameter optimization [11]. Although such systems, once developed, can become widely accessible, they still involve several critical limitations. As these models are typically trained on specific datasets, they may encounter issues with generalizability across different populations, require continuous technical intervention for updates and retraining with new data, and necessitate the installation of specialized infrastructure within clinical settings. In this context, the present study aims to evaluate the effectiveness of chatbot systems—LLM-based artificial intelligence models—in predicting bone age and identifying the pubertal growth stage, while also exploring their potential as a flexible, cost-effective, and rapid alternative within clinical decision support systems. This study also explored the effectiveness of AI-based ChatGPT models that can directly analyze hand–wrist radiographs to predict skeletal bone age and growth stages without the need for pre-trained, domain-specific image processing algorithms. Furthermore, we investigated whether this infrastructure-independent approach could be a viable alternative to traditional image analysis techniques and specialized medical AI systems. The null hypothesis of the study is that AI-based GPT models do not significantly differ in accuracy in their ability to predict bone age and growth stage from reference data.

## 2. Materials and Methods

Ethical approval for this study was obtained from the Non-Interventional Clinical Research Ethics Committee of Zonguldak Bülent Ecevit University (Date: 9 April 2025; Protocol No: 2025/07-11). A total of 90 left hand–wrist radiographs, originally acquired from patients who were referred for orthodontic treatment at the Department of Orthodontics, Faculty of Dentistry, Zonguldak Bülent Ecevit University, were collected. All radiographs were captured using an X-ray device (Veraviewepocs 2D, J. Morita Mfg. Corp., Kyoto, Japan) at a voltage of 80 kV, an exposure time of 5.0 s, 3.0 mA, and a dose area product (DAP) of 3.74 mGy·cm^2^. All participants and their legal guardians signed a written informed consent form for the inclusion of their hand–wrist radiographic images in the study. Due to the retrospective nature of the study, no additional consent was obtained.

The study included high-resolution hand–wrist radiographs that clearly showed the anatomical structures of each growth stage and were free of imaging artifacts or other defects. Radiographs that did not meet at least one of these criteria were excluded from the study. To ensure anonymization, all numerical and textual information related to patient identification was cropped from the radiographs, resulting in randomized hand–wrist images. Thus, the chatbots were prevented from accessing any chronological age information embedded in the radiographic images, thereby eliminating potential cues related to the patient’s actual age.

A total of 90 hand–wrist radiographs were evaluated by two orthodontists, each with a minimum of five years of clinical experience in assessing bone age and growth–development stages. The evaluators classified each radiograph into one of three growth stages—pre-peak, peak, or post-peak—based on the criteria defined in Fishman’s Skeletal Maturity Indicators (SMI) system. For each growth stage, 15 radiographs from female patients and 15 from male patients were selected, resulting in a total of 30 radiographs per stage and 90 radiographs included in the study. Subsequently, the evaluators determined the bone age of each patient using the Greulich–Pyle Atlas as a reference. The bone age ranges identified for each group were as follows: 8.16 to 12.15 years for the pre-peak group, 10.00 to 15.33 years for the peak group, and 12.06 to 16.00 years for the post-peak group. Inter-rater reliability for bone age prediction was assessed and found to be high (ICC = 0.927), indicating strong consistency between the two evaluators [24]. In instances of disagreement, a consensus was reached through discussion. If consensus could not be achieved, a third orthodontist with over ten years of professional experience was consulted to provide an independent evaluation and resolve the discrepancy. In this way, each hand–wrist radiograph was divided according to the growth–development stage based on the Fishman Skeletal Maturity Indicators (SMI) system, and bone age was determined for each image using the Greulich–Pyle Atlas as a reference. These assessments were used to establish the gold-standard reference values for the study.

Three AI-based ChatGPT models developed by OpenAI (San Francisco, CA, USA) were used in the study through a ChatGPT Pro subscription: GPT-4o (the model introduced by OpenAI as capable of real-time reasoning across audio, vision, and text) [25], GPT-o4-mini-high (the model introduced by OpenAI as demonstrating exceptional performance, particularly in tasks involving mathematics, coding, and visual reasoning) [26], and GPT-o1-pro (the model that is said to demonstrate superior performance in challenging machine learning benchmarks in mathematics, science, and coding) [27]. The hand–wrist radiographs categorized into each growth stage were individually uploaded to three AI-based ChatGPT models. Each image was submitted with the following prompt: “*You are an orthodontist. This is a hand-wrist radiograph of a male/female patient. Please evaluate the epiphyseal-diaphyseal relationship based on the Greulich-Pyle atlas and predict the bone age in terms of months and years*.” Following the initial response, a second prompt was sent to the same platform: “*Now, please indicate which of the growth and development stages (pre-peak, peak, or post-peak) this hand-wrist radiograph corresponds to, based on Fishman’s Skeletal Maturity Indicators (SMI)*.” In this manner, both bone age predictions and growth stage classifications were obtained for each uploaded radiograph. After every individual evaluation, the chat history was cleared, and a new window was opened to submit the next radiograph using the same prompt, ensuring no contextual memory influenced subsequent responses. All interactions were performed by a single researcher in May 2025 using the same laptop (MacBook Air M3, 16 GB RAM; Apple, Cupertino, CA, USA), a 4.5G internet connection, and a virtual private network (VPN) server (version 3.9; Astrill Systems Corp., Santa Clara, CA, USA). Each radiograph, along with its corresponding prompts, was submitted to each AI model a single time. No clarification or follow-up was provided in cases where the model failed to generate a response. All submissions were carried out by the same researcher to ensure consistency, and no prompts were repeated or rephrased by a second individual. The bone age predictions and the predicted growth–development stages generated by the chatbot models were recorded.

### Statistical Analysis

The statistical analyses were performed using SPSS (version 26, IBM Corporation, New York, NY, USA) and Python (version 3.11.2; Python Software Foundation, Beaverton, OR, USA) software. To evaluate the classification performance of the models across different growth–development stages, Cohen’s Kappa analysis was conducted separately for each class (pre-peak, peak, and post-peak). The agreement between bone age predictions generated by the AI models and the gold-standard values determined by expert raters was assessed using the Pearson Correlation Coefficient. The mean absolute error (MAE) was calculated to quantify the average deviation of the models’ predictions from the reference values. To examine the differences in mean MAE values across the three models, a repeated measures ANOVA was performed. In order to investigate whether the individual differences between the AI-generated predictions and the reference bone ages contained systematic bias and whether these differences remained within acceptable limits, a Bland–Altman analysis was conducted. In the Bland–Altman plots, the differences between the predicted and reference values were plotted against the mean of each prediction–reference pair, allowing the position of each measurement pair to be visualized on the graph. The analyses involved the evaluation of the mean difference (bias), the limits of agreement defined as ±2.58 standard deviations from the mean difference, and the regression relationship between the differences and the means. The *p*-value was considered statistically significant at less than 0.05. Also, to evaluate the classification performance of the models, accuracy, sensitivity, precision, specificity, and F1 score were calculated. Accuracy represents the overall proportion of correctly classified cases ((TP + TN)/(TP + TN + FP + FN)). Specificity measures the ability to correctly identify negative cases (TN/(TN + FP)). Precision indicates the proportion of predicted positive cases that are truly positive (TP/(TP + FP)), where TP is the number of true positives and FP is the number of false positives. Sensitivity reflects the model’s ability to correctly identify actual positive cases (TP/(TP + FN)), where FN is the number of false negatives. F1 score is the harmonic mean of precision and recall, providing a balanced measure of the model’s performance (F1 = 2 × (Precision × Sensitivity)/(Precision + Sensitivity)) [8].

## 3. Results

A statistically significant association was found between the GPT-4o model’s predictions and the reference classifications for the pre-peak and post-peak growth periods. The Cohen’s Kappa coefficient was calculated as 0.283, indicating a low but statistically significant level of agreement between the GPT-4o model and the reference classifications (*p* < 0.001). In contrast, the comparison between the GPT-o4-mini-high model and the reference classifications yielded a Cohen’s Kappa value of 0.133, which was not statistically significant (*p* = 0.073). Similarly, the GPT-o1-pro model demonstrated a Cohen’s Kappa value of 0.117 in comparison with the reference classifications, which also did not reach statistical significance (*p* = 0.095). The results of the Cohen’s Kappa analysis assessing the agreement between the growth–development stage predictions of the GPT-4o, GPT-o4-mini-high, and GPT-o1-pro chatbot models and the reference classifications are presented in Table 2.

When the performance metrics were reviewed, the GPT-4o model achieved the highest overall accuracy across all stages (0.522), standing out particularly in the post-peak stage with a specificity of 0.950. The GPT-o4-mini-high model demonstrated high specificity (0.833) in the pre-peak stage, while the GPT-o1-pro model exhibited high specificity (0.850) in the post-peak stages. Table 3 presents the performance metrics (accuracy, sensitivity, specificity, precision, and F1 score) of the three chatbot models (GPT-4o, GPT-o4-mini-high, and GPT-o1-pro) in identifying the pre-peak, peak, and post-peak growth stages.

To compare the bone age prediction accuracy of the three AI models, a repeated measures ANOVA was conducted on their mean absolute error (MAE) values. According to descriptive statistics, the mean MAE was 2.03 years (SD = 2.26) for the GPT-4o model, 1.54 years (SD = 1.42) for the GPT-o4-mini-high model, and 1.57 years (SD = 1.20) for the GPT-o1-pro model. Pairwise comparisons with Bonferroni correction revealed no statistically significant differences among the models (*p* > 0.05).

**Figure 1:**
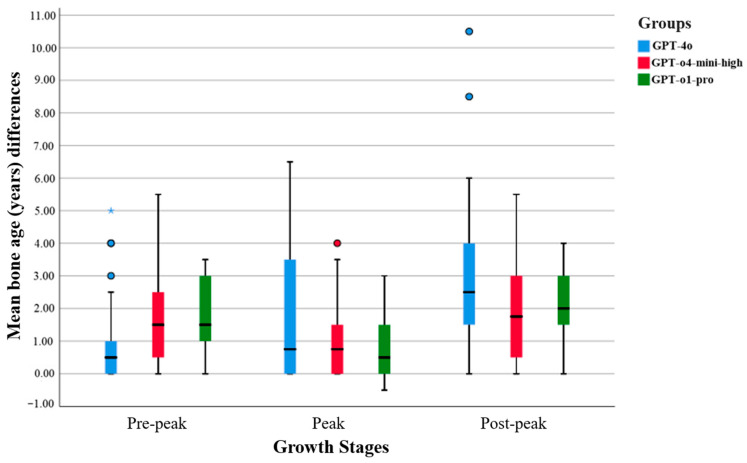
Clustered box plot of mean bone age (years) differences in chatbots by growth stages. Extreme outliers in the data are indicated by an asterisk (☆) and circles (○). The black line within the box denotes the median value, representing the central tendency of the data distribution.

The GPT-o1-pro model demonstrated narrower interquartile ranges, fewer outliers, and lower mean differences across all growth stages, indicating more consistent and less variable predictions. In contrast, the GPT-4o model exhibited broader distributions and a greater number of outliers, reflecting increased variability in its predictions. The GPT-o4-mini-high model generally fell between the two in terms of performance, but occasionally displayed higher variance levels (see Figure 1).

According to the results of the Pearson correlation analysis, the highest positive correlation with the reference bone age values was observed in the GPT-o1-pro group (r = 0.546, *p* < 0.001), followed by the GPT-o4-mini-high group (r = 0.491, *p* < 0.001) and the GPT-4o group (r = 0.395, *p* < 0.001). A statistically significant correlation was also found between the GPT-4o and GPT-o1-pro groups (r = 0.503, *p* < 0.001). However, the correlation between the GPT-4o and GPT-o4-mini-high groups was not statistically significant (r = 0.156, *p* = 0.143). These findings indicate that all chatbot models demonstrated statistically significant positive correlations with the reference values. Among them, the GPT-o1-pro model showed the strongest correlation, suggesting that its bone age predictions were moderately correlated with the reference data and were not due to chance. The detailed results of the Pearson correlation analysis are presented in Table 4.

The prediction accuracies of the three chatbot models within ±1 year, ±1.5 years, and ±2 years deviation range from the reference bone ages are presented in Table 5.

**Figure 2:**
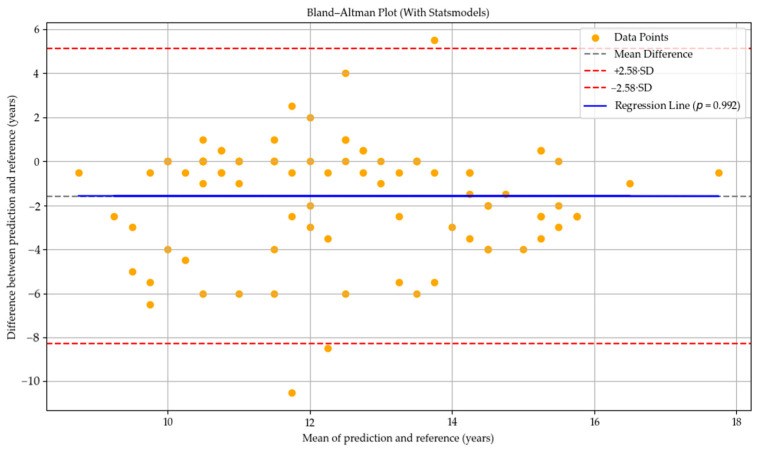
Bland–Altman plot illustrating the agreement between bone age predictions by GPT-4o and the reference values. The black dashed line indicates the mean difference (bias), and the red dashed lines represent the 99% limits of agreement (±2.58 SD). The blue regression line shows no significant proportional bias (*p* = 0.992). Yellow dots represent individual data points.

In the Bland–Altman plot for the GPT-4o model, most of the prediction–reference differences are located in the negative region, and the mean difference line lies below zero. This indicates a general tendency of the GPT-4o model to underpredict bone age compared to the reference values. However, the blue regression line is nearly horizontal and does not exhibit a statistically significant slope (*p* = 0.992), suggesting that the model’s deviations do not systematically vary with the average bone age. Therefore, no evidence of proportional bias related to age was observed (see Figure 2).

**Figure 3:**
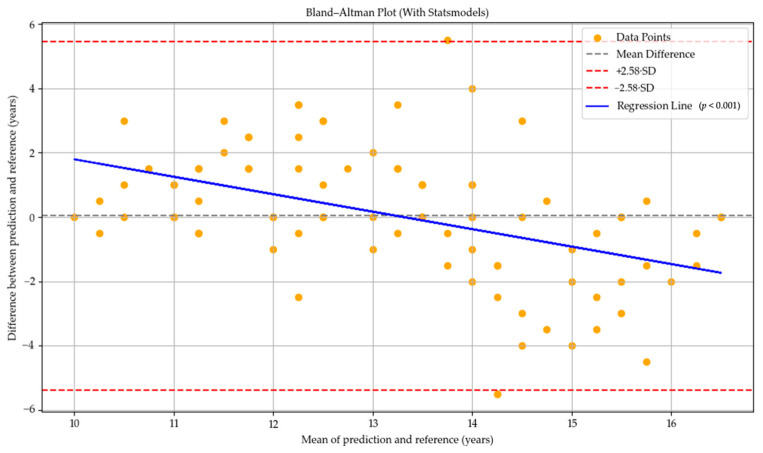
Bland–Altman plot illustrating the agreement between bone age predictions by GPT-o4-mini-high and the reference values. The black dashed line indicates the mean difference (bias), and the red dashed lines represent the 99% limits of agreement (±2.58 SD). The blue regression line shows a significant proportional bias (*p* < 0.001). Yellow dots represent individual data points.

In the GPT-o4-mini-high model, the mean difference line was positioned very close to zero, indicating a general tendency toward more balanced predictions. This finding suggests that the model tended to overpredict bone age in individuals at earlier stages of skeletal development, while underpredicting it in those at more advanced stages. The regression line exhibited a statistically significant negative slope (*p* < 0.001), indicating the presence of an age-related proportional bias in the model’s predictions (see Figure 3).

**Figure 4:**
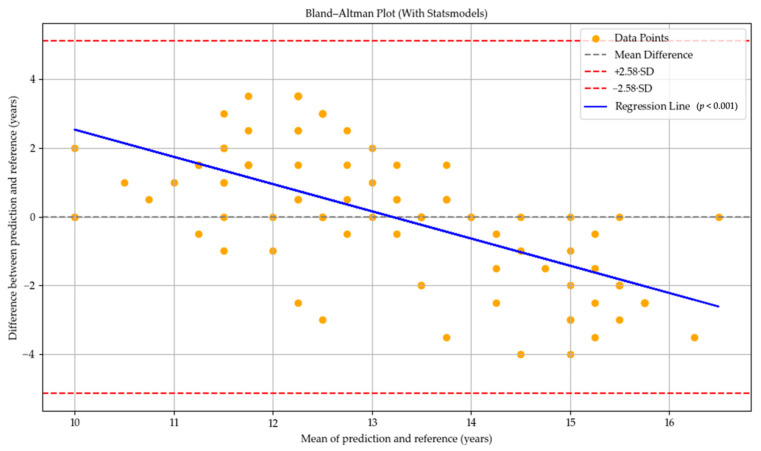
Bland–Altman plot illustrating the agreement between bone age predictions by GPT-o1-pro and reference values. The black dashed line shows the mean difference (bias), while red dashed lines represent the 99% limits of agreement (±2.58 SD). The blue regression line indicates a significant proportional bias (*p* < 0.001). Yellow dots represent individual observations.

In the Bland–Altman plot for the GPT-o1-pro model, the mean difference was located in the positive region, indicating a general tendency of the model to overpredict bone age compared to the reference values. However, the regression line displayed a clear negative slope, which was found to be statistically significant (*p* < 0.001). This suggests the presence of a proportional bias related to the average bone age, whereby the model tended to overpredict bone age in individuals at earlier developmental stages and underpredict it in those at more advanced stages (see Figure 4).

## 4. Discussion

Bone age assessment is inherently time-consuming and subject to considerable inter-observer variability, posing significant challenges in clinical contexts where treatment decisions rely on the evaluation of skeletal maturity changes [11]. Consequently, the application of artificial intelligence systems for predicting bone age from hand–wrist radiographs has attracted growing interest in recent years [11,13,14,15,16]. The present study evaluated the performance of AI-based ChatGPT models in predicting bone age and growth stages using hand–wrist radiographs.

Although previous studies have demonstrated that LLMs such as ChatGPT exhibit strong performance in text-based tasks, their capabilities in image interpretation remain comparatively limited [8,28,29]. In some studies suggesting that LLMs hold high potential in ultrasonographic analysis, the models did not directly interpret medical images; instead, conclusions were drawn based on the interpretation of text-based ultrasound reports [30,31]. Consequently, since LLMs are predominantly trained on textual data, they tend to perform better in tasks such as natural language understanding, logical reasoning, and the generation of structured reports. This allows them to make more accurate predictions based on information that has been transformed into text format, rather than through direct pixel-level analysis. The primary reason behind the limited performance of current LLM architectures in image analysis is that these models are inherently designed for text-based data. Their inability to process visual information at the pixel level, interpret spatial relationships, and grasp anatomical structures hinders their capacity to analyze radiographic images—particularly in tasks that require the detection of subtle grayscale nuances, edge sharpness, and textural variations [32].

In the present study, the bone age prediction performance of the chatbot models was evaluated, revealing that the GPT-4o model achieved an accuracy rate of up to 53% within a ±1 year deviation range, while the GPT-o4-mini-high model reached an accuracy of up to 72.2% within a ±2 year deviation range. While the models demonstrate limited overall predictive accuracy, the observed deviations fall within clinically acceptable limits when benchmarked against the error margins of traditional bone age assessment techniques reported in the literature. Previous studies comparing the Greulich–Pyle method with alternative manual bone age assessment techniques have demonstrated that discrepancies between methods may commonly reach up to 1–2 years, highlighting the considerable variability that can arise depending on the assessment approach [33,34].

Based on the findings, while the GPT-4o model demonstrated a higher mean absolute error (MAE = 2.03, SD = 2.26) compared to the other models, this difference was not statistically significant (*p* > 0.05). These results suggest that, although the chatbot models exhibit similar ranges of error in bone age prediction, further analyses reveal that there are discernible performance differences among the individual models.

Correlation analyses conducted between the chatbot model predictions and the reference measurements revealed that all models exhibited statistically significant linear relationships with the reference values (*p* < 0.001). These findings indicate that the predictions generated by the models were not random and demonstrated a certain degree of alignment with the reference standards, particularly highlighting the GPT-o1-pro model as producing more consistent results. Specifically, the correlation between the GPT-4o model and the reference values was weak (r = 0.395), the correlation for the GPT-o4-mini-high model was between weak and moderate (r = 0.491), and the GPT-o1-pro model demonstrated a moderate correlation (r = 0.546) [24]. A previous study conducted by Zhu et al. [8] reported that the GPT-4o model demonstrated low consistency in medical image classification tasks. In contrast, the fact that the present study was conducted more recently (May 2025) is noteworthy, as it may reflect ongoing improvements in GPT-based models over time, suggesting a progressive enhancement in their performance, particularly in tasks involving visual diagnostic interpretation.

Ren et al. [35] reported that GPT-4o was effective in responding to general diagnostic questions, such as “Is there a lesion in this osteosarcoma X-ray?”, and was capable of identifying obvious bone lesions. However, the model demonstrated low sensitivity in making specific diagnostic determinations. Similarly, another study found that while ChatGPT-4o exhibited high accuracy in detecting the presence of knee osteoarthritis, its performance was considerably lower when tasked with classifying the severity or stage of the disease [8]. These findings suggest that while the model may be functional for general diagnostic tasks, it remains limited in its ability to perform detailed clinical differentiation. A similar pattern was observed in the LLM-based GPT models evaluated in the present study. Although some models demonstrated relatively good alignment with the reference values in bone age prediction, their performance was more limited when it came to the precise classification of growth stages. Specifically, the GPT-4o model correctly classified 14 of the peak-phase radiographs (46.6%), the GPT-o4-mini-high model correctly classified 9 (30%), and the GPT-o1-pro model correctly identified 17 (56.6%). Considering that accurate identification of the peak growth stage is critical for initiating timely orthodontic intervention, particularly in cases requiring early treatment [11], the findings of this study suggest that caution should be exercised in clinical decision-making, especially when utilizing the GPT-o4-mini-high model. Nonetheless, the GPT-o1-pro model achieved a classification accuracy of 56.6% in identifying one of the three possible growth stages, significantly exceeding the 33.3% accuracy rate expected by chance. This suggests that the model demonstrates a level of pattern recognition beyond random selection and may hold potential for further development in growth stage classification tasks. Accordingly, the null hypothesis of the study was rejected due to differences between the GPT models’ bone age and growth stage predictions and the reference data.

In a recent study investigating the performance of chatbot models in classifying thyroid nodules as benign or malignant based on ultrasonographic images, the models were also evaluated in terms of their ability to accurately determine the necessity of biopsy. According to the results, in 41.4% of the cases where the ChatGPT-4o model recommended a biopsy, no clinical indication for biopsy was present [32]. In alignment with these results, our study also observed that GPT-based models frequently misclassified radiographs of post-peak patients as belonging to the pre-peak or peak stages. This finding raises concerns that, at their current level of performance, the models may potentially recommend inappropriate early treatment options for patients whose growth and development phases have already concluded.

Although the GPT-4o model exhibited a lower correlation with reference values in bone age prediction compared to the other models, the Bland–Altman analysis indicated that it did not present a systematic bias across age groups. While this suggests that GPT-4o may produce more consistent and predictable results, the wide range of differences between its predictions and the reference values indicates that the model may still yield considerable variation in individual cases. In contrast, the GPT-o4-mini-high and GPT-o1-pro models exhibited a systematic bias in which the predicted bone age tended to fall below the reference values as chronological age increased. This suggests that the models in question may have a limited ability to account for skeletal changes that occur with increasing age. In the Bland–Altman plots, particularly in models exhibiting systematic bias (see Figure 2 and Figure 3), the models’ tendency to overestimate bone age may potentially result from their inability to distinguish anatomical features characteristic of early developmental stages from those indicative of more advanced skeletal maturation. Conversely, the underestimation of age in older individuals could be explained by the models’ limited ability to accurately detect fine details such as fusion lines in the epiphyseal–diaphyseal regions, potentially interpreting all signs of fusion as a definitive endpoint of development. This suggests that the models may regard certain morphological thresholds as indicators of maturation completion. In the Bland–Altman plot of the GPT-o1-pro model (see Figure 4), narrower ranges of deviation were observed compared to the other models, indicating that its errors were more predictable and consistent. As observed in the study, each model displays distinct limitations depending on the aspect under evaluation. This highlights the current challenge faced by LLMs, which are fundamentally text-based, in their ability to effectively analyze radiographic structures. Another important limitation is the need for larger and multi-center datasets to support the external validity of the models. This limitation may be overcome in future models through targeted training on medical imaging data and the adoption of advanced multimodal architectures that effectively integrate visual and textual information, thereby enabling more accurate clinical interpretation [32].

CNN-based bone age prediction methods have demonstrated significant success in this field due to their ability to learn directly from image data and provide highly accurate age predictions. These systems can analyze the morphological characteristics of bone structures in hand–wrist radiographs in detail and typically achieve accuracy rates exceeding 90% within a ±1 year deviation range [11,13]. In their study, Lee et al. [11] reported that the CNN-based bone age assessment system they developed achieved an accuracy rate of 98.11% to 99% within a 2-year margin of error. Van Rijn et al. [16] also found the accuracy rate to be close to 90% within a 2-year margin of error. According to the findings of our study, although LLM-based ChatGPT systems have not yet achieved the same level of accuracy in bone age prediction as CNN-based models, they offer significant advantages over traditional CNN approaches. To the best of our knowledge, this is the first computer-aided analysis of hand–wrist radiographs using AI-based ChatGPT models in the literature, performed without prior training on large datasets, with the aim of emphasizing these advantages. LLM-based systems are not limited to image analysis; they can also interpret contextual information provided through natural language inputs, allowing for a more comprehensive understanding of the patient’s condition. This capability becomes particularly valuable in complex clinical scenarios. By analyzing imaging data in conjunction with clinical findings, LLM-based systems enable more comprehensible interpretations and facilitate clinician–patient communication. Additionally, due to their interactive and easy-to-use design, LLM systems can be rapidly integrated into healthcare environments without the need for specialized infrastructure or high-cost hardware. To enhance the diagnostic accuracy of LLM models in the future, it will be essential to train them through task-specific fine-tuning using medical imaging datasets [8] and to architect them for seamless integration with multimodal systems. A hybrid diagnostic workflow in which a system like ChatGPT conducts an initial evaluation that is subsequently reviewed and validated by a dental or medical expert has substantial potential to improve clinical reliability and operational efficiency [8].

## 5. Conclusions

Although general-purpose vision–language models do not yet offer a complete solution for radiographic diagnostics, they represent a promising starting point. Based on our findings, GPT-4o demonstrated more consistent outputs without systematic bias, GPT-o4-mini-high achieved the highest accuracy within a ±2 year deviation range, and GPT-o1-pro showed the strongest correlation with the reference values. These results highlight that each model possesses distinct strengths in bone age prediction. This suggests that the models exhibit complementary characteristics, and a combined model approach may offer more balanced and reliable predictions. The study findings point to the future potential of LLM-based chatbot models, indicating that their use in preliminary evaluation processes may meaningfully contribute to early diagnosis and clinical decision-making. At present, it appears more appropriate to position these models as tools that support clinicians rather than as independent decision-makers. Nonetheless, the ability of GPT models to achieve this level of performance without domain-specific medical training could be recognized as a significant conceptual achievement.

## Figures and Tables

**Table 1:** Studies employing different machine learning techniques and their performance metrics.

Machine Learning Techniques	Study (Author, Year)	Dataset Size	Performance
Regression-based methods	Thodberg et al. (2009) [15]	1559	MSE: 0.42–0.80 (year)
Artificial Neural Networks	Lin et al. (2012) [17]	600	MSE: 0.26 (classification error rate)
	Tang et al. (2018) [18]	79	Mean disparity (year): 0.13 (male) and 0.08 (female)
Convolutional Neural Networks	Lee et al. (2017) [11]	8325	Accuracy (± 2 years): 99.00% (male) and 98.11% (female)
	Kim et al. (2023) [19]	21,036	MAE: 6.1 (month)
Support Vector Machines	Haak et al. (2013) [20]	1097	RMS: 0.73 (year)

MAE: mean absolute error; MSE: mean squared error; RMS: root mean square.

**Table 2:** Statistical analysis results of Cohen’s Kappa values for GPT groups based on growth periods.

Chatbot Model	Growth Period	References			Cohen’s Kappa Value	*p*
		Pre-Peak	Peak	Post-Peak		
		*n* (%)	*n* (%)	*n* (%)		
GPT-4o	Pre-peak	22 (51.2) ^a^	7 (21.2) ^b^	1 (7.1) ^b^	0.283	<0.001 *
	Peak	14 (32.6) ^a^	14 (42.4) ^a^	2 (14.3) ^a^		
	Post-peak	7 (16.3) ^a^	12 (36.4) ^a^	11 (78.6) ^b^		
	Total	43 (100)	33 (100)	14 (100)		
GPT-o4-mini-high	Pre-peak	17 (63)	7 (24.1)	6 (17.6)	0.133	0.073
	Peak	5 (18.5)	9 (31)	16 (47.1)		
	Post-peak	5 (18.5)	13 (44.8)	12 (35.3)		
	Total	27 (100)	29 (100)	34 (100)		
GPT-o1-pro	Pre-peak	14 (56)	13 (26)	3 (20)	0.117	0.095
	Peak	7 (28)	17 (34)	6 (40)		
	Post-peak	4 (16)	20 (40)	6 (40)		
	Total	25 (100)	50 (100)	15 (100)		

*n*: sample; %: percentage; *p*: significance level; *: *p* < 0.05; ^a,b^: there is a statistically significant difference between groups with different top index letters in the same row.

**Table 3:** Performance metrics of three LLM-based chatbot models in classifying skeletal growth stages (pre-peak, peak, and post-peak).

Chatbot Model	Accuracy	Growth Period	Sensitivity	Specificity	Precision	F1 Score
GPT-4o	0.522	Pre-Peak	0.733	0.650	0.512	0.603
		Peak	0.467	0.683	0.424	0.444
		Post-Peak	0.367	0.950	0.786	0.498
GPT-o4-mini-high	0.422	Pre-Peak	0.567	0.833	0.630	0.597
		Peak	0.300	0.667	0.310	0.305
		Post-Peak	0.400	0.633	0.353	0.375
GPT-o1-pro	0.411	Pre-Peak	0.467	0.817	0.560	0.509
		Peak	0.567	0.450	0.340	0.426
		Post-Peak	0.200	0.850	0.400	0.267

**Table 4:** Correlation analysis results between the reference group and the GPT groups.

Group		Reference	GPT-4o	GPT-o4-mini-high	GPT-o1-pro
**Reference**	Pearson r	1	0.395	0.491	0.546
	*p*		<0.001 *	<0.001 *	<0.001 *
**GPT-4o**	Pearson r		1	0.156	0.503
	*p*			0.143	<0.001 *
**GPT-o4-mini-high**	Pearson r			1	0.295
	*p*				0.005 *
**GPT-o1-pro**	Pearson r *p*				1

*p*: significance level, *: *p* < 0.05.

**Table 5:** Bone age prediction accuracy (%) of three GPT models at different deviation ranges (≤1 year, ≤1.5 years, and ≤2 years).

	≤1 Year	≤1.5 Year	≤2 Year
**GPT-4o**	53.3	54.4	60
**GPT-o4-mini-high**	50.0	65.5	72.2
**GPT-o1-pro**	43.3	57.7	66.6

Values indicate the percentage of predictions within the specified range from the reference bone age.

## Data Availability

Data are entirely contained within the article.
